# Genotypic and Phenotypic Investigation of Clinical *Aspergillus* isolates from Iran Indicates Nosocomial Transmission Events of *Aspergillus flavus*

**DOI:** 10.1007/s11046-025-00988-w

**Published:** 2025-08-30

**Authors:** Bram Spruijtenburg, Sadegh Khodavaisy, Jacques F. Meis, Theun de Groot, Jianping Xu, Sayed Jamal Hashemi, Mohammadreza Salehi, Zeinab Borjian Boroujeni, Farzad Aala, Ali Ahmadi, Sareh Montazeri, Jezreel Dalmieda, Eelco F. J. Meijer

**Affiliations:** 1 Department of Medical Microbiology, Radboudumc, Nijmegen, The Netherlands; 2 Radboudumc-CWZ Center of Expertise for Mycology, Nijmegen, The Netherlands; 3 Department of Medical Microbiology and Immunology, Canisius-Wilhelmina Hospital (CWZ)/Dicoon, Nijmegen, The Netherlands; 4 Department of Biology, McMaster University, Hamilton, ON Canada; 5 Department of Medical Parasitology and Mycology, School of Public Health, Tehran University of Medical Sciences, Tehran, Iran; 6 Research Center for Antibiotic Stewardship and Antimicrobial Resistance, Tehran University of Medical Sciences, Tehran, Iran; 7 Institute of Translational Research, Cologne Excellence Cluster on Cellular Stress Responses in Aging-Associated Diseases (CECAD) and Excellence Center for Medical Mycology, University of Cologne, Cologne, Germany; 8 Department of Parasitology and Mycology, Faculty of Medicine, Kurdistan University of Medical Sciences, Sanandaj, Iran; 9 Department of Medical Parasitology and Mycology, School of Medicine, Shahid Beheshti University of Medical Sciences, Tehran, Iran; 10 Department of Parasitology and Mycology. School of Medicine, Zabol University of Medical Sciences, Zabol, Iran

**Keywords:** Aspergillosis, Antifungal resistance, Genotyping, Outbreak

## Abstract

Aspergillosis is one of the most common human fungal infections. The invasive form of this infectious disease has high mortality rates. Moreover, antifungal resistance has been increasing, thereby limiting treatment options. In Iran, limited species distribution, genotyping and susceptibility data regarding aspergillosis is available, especially during the COVID-19 pandemic. In the current study, 124 patients with proven (n = 31), probable (n = 24) and possible (n = 46) aspergillosis and aspergillus colonization (n = 19) were investigated. Isolates were identified to species level based on calmodulin sequencing. Antifungal susceptibility testing was performed with microbroth dilution against common antifungal agents, i.e. amphotericin B, azoles and echinocandins. Additionally, short tandem repeat genotyping was conducted on common *Aspergillus* species to assess genetic relatedness. *Aspergillus flavus* was the most common species for proven aspergilossis cases, followed by identification of single cases of *A. fumigatus*, *A. terreus*, *A. niger*. These *Aspergillus* species were also mostly found in other patients with probably aspergillosis, in addition to four cases of possible aspergillosis with rare or cryptic species *A. candidus*, *A. citrinoterreus*, *A. tubingensis* and *A. fumigatiaffinis*, respectively. Using available epidemiological cutoff values (ECVs) no isolates were non-wild type to the tested antifungal drugs, while the *A. fumigatiaffinis* and *A. citrinoterreus* isolate demonstrated reduced susceptibility to respectively amphotericin B and Itraconazole, and amphotericin B only. With high-resolution short tandem repeat genotyping, several *A. flavus* clusters were found and their spatial and temporal clustering suggested nosocomial origins. To conclude, aspergillosis cases in Iran were caused by diverse but susceptible species, with *A. flavus* being dominant and associated with several events of potential nosocomial transmission.

**Supplementary Information:**

The online version contains supplementary material available at 10.1007/s11046-025-00988-w.

## Introduction

Invasive fungal infections (IFI) pose a significant threat among immunocompromised individuals, resulting in high mortality [1]. Aspergillosis is one of the most common filamentous fungal infections with increasing prevalence. *Aspergillus* species, particularly *A. fumigatus*, *A. flavus*, and *A. terreus*, are involved in various forms of human disease and affect both immunocompetent and immunocompromised subjects. COVID-19-associated pulmonary aspergillosis (CAPA) is a subtype of aspergillosis that causes infection in the lungs of critically ill COVID-19 patients [2]. The incidence of CAPA among COVID-19 patients in intensive care units (ICUs) has been reported to range from 2.5% to 35%, with high mortality rates of up to 60% [3]. Other forms of the disease include chronic pulmonary aspergillosis (CPA) and invasive aspergillosis (IA), with patients presenting with diverse risk factors like immunotherapy and leukemia [3].

With an increase in susceptible human host populations and the emergence of drug resistance in fungal pathogens, there is an urgent need to manage these life-threatening infections [1]. Genotyping methods have been employed in many countries to understand the diversity and epidemiology among fungal pathogens in infected patients, but there is currently limited information about these fungal infections in Iranian clinical samples [4]. This study aims to genetically identify *Aspergillus* species of clinical isolates from Iran, determine antifungal susceptibility, and genotype isolates using short tandem repeat (STR) markers [5], to reveal the genetic diversity and the potential relatedness between these strains.

## Material and Methods

### Study Design and Patient Data

This was a prospective observational study during the COVID-19 pandemic from July 2020 to October 2023 conducted at Tehran University-affiliated teaching hospitals. Patients with proven, probable or possible of aspergillosis according to EORTC/MSGERC displaying at least one of the following host factors were enrolled in the study: hematologic malignancy; cancer and receiving chemotherapy with or without neutropenia; chronic obstructive pulmonary disease (COPD); cystic fibrosis, transplant recipient; other immunocompromised state (inherited immunodeficiency, child C cirrhosis, or HIV, etc.); COVID-19; recipient of any other immunosuppressive treatment (tacrolimus, cyclosporine, cyclophosphamide, etc.) [6]. Clinical data including demographics, underlying diseases, and clinical features were collected. Different specimens, such as bronchoalveolar lavage (BAL), endotracheal tube secretions, sputum, synovial fluid, wound and tissue were collected, transported to and processed at the Medical Mycology Laboratory. The study was conducted according to the guidelines of the Declaration of Helsinki [7], and the study was approved by the ethics committee of Tehran University of Medical Sciences (Ref no. IR.TUMS.SPH.REC.1401.286). Isolates were assigned with numerical codes to anonymize the patient’s identity.

### Molecular Identification

Prior to identification, isolates were grown on Sabouraud dextrose agar (SDA) with chloramphenicol (100 mg/L) for seven days at 35°C. DNA extraction and purification was performed as described earlier [8]. In short, harvested mycelia from isolates were lysed with MagNA Pure bacteria lysis buffer and MagNA Lyser green bead (Roche Diagnostics GmbH, Mannheim, Germany). DNA extraction was performed with the MagNA Lyser system and the MagNA Pure Viral NA Small Volume Kit according to manufacturer’s instructions (Roche diagnostics). The calmodulin (*CaM*) gene was amplified with PCR using primers Cmd5 5’-CCGAGTACAAGGARGCCTTC-3' and Cmd6 5'-CCGATRGAGGTCATRACGTGG-3' [9]. PCR according to the BrilliantDye (Nimagen, Nijmegen, The Netherlands) protocol, purifications with Ampliclean and D-Pure purification (both Nimagen) and subsequent Sanger sequencing on a 3500 XL genetic analyzer (Applied Biosystems, Foster City, CA, USA) were performed as previously described [10]. *CaM* sequences of *Aspergillus* reference strains were retrieved from the National Center for Biotechnology (NCBI) nucleotide database (Table S1). Sequence alignment and visualization were performed using MAFFT v7 and IQ-TREE web server as described earlier in detail [10]. All sequences generated in the present study were deposited under GenBank accession numbers PP350317-PP350410 and PP795459-PP795492 (Table S2).

### Antifungal Susceptibility Testing (AFST)

In vitro AFST on all isolates was performed with broth microdilution according to CLSI M38 3rd edition guidelines [11]. The drug concentration range of 0.016–16 µg/mL was used for amphotericin B (Bristol Meyers Squibb, Woerden, The Netherlands), voriconazole (Pfizer Central Research, Sandwich, UK), itraconazole (Janssen Cilag, Beerse, Belgium), posaconazole (Merck, Haarlem, The Netherlands) and isavuconazole (Basilea Pharmaceutica, Basel, Switzerland) and the range of 0.008–8 µg/mL was used for micafungin and anidulafungin (both from Merck). Minimum inhibitory concentrations (MICs) were read visually after 48 h of incubation at 35 °C as the lowest concentration with a 100% growth reduction, while minimum effective concentrations (MECs) for the echinocandins were read microscopically as the lowest concentration at which short, stubby and highly branched hyphae were observed. Epidemiological cutoff values (ECVs) of the CLSI M59 document were implemented to classify isolates as wild type or non-wild type [12].

### Short Tandem Repeat (STR) Genotyping

Genotyping for *A. flavus*, *A. fumigatus* and *A. terreus* was performed as previously described [13–15]. Multiplex PCRs amplifying nine STR markers for each species were performed using 1 × FastStart *Taq* polymerase buffer without MgCl_2_, deoxynucleoside triphosphates (dNTPs) (0.2 mM), MgCl_2_ (3mM), forward and reverse primers (5 µM), 1 U FastStart *Taq* polymerase (Roche Diagnostics), and isolated DNA on a thermocycler (Biometra, Jena, Germany). Products were analyzed on a 3500 XL genetic analyzer (Applied Biosystems) and resulting copy numbers of repeating nucleotide units within the marker loci were determined and analyzed using Genemapper 5 (Applied Biosystems) and BioNumerics v7.6.1 (Applied Matchs NV, Sint-Martens-Latem, Belgium) as described before [8].

## Results

### Patient Characteristics and Risk Factors

A total of 127 *Aspergillus* isolates were collected from 124 patients with 31 proven, 24 probable, 46 possible and 19 colonization cases, according to EORTC/MSGERC criteria (Table S2). For patients with proven aspergillosis common sample specimen included tissue (19%), sputum, bronchoalveolar lavage and nasal biopsies (all three 13%). Genders were equally distributed with 52% male, and many were admitted to the general ward (29%). The main underlying condition was COVID-19 (42%). From two proven and one probable aspergillosis cases, two isolates were recovered on the same day from different sites.

### Molecular Species Identification

**Fig. 1:**
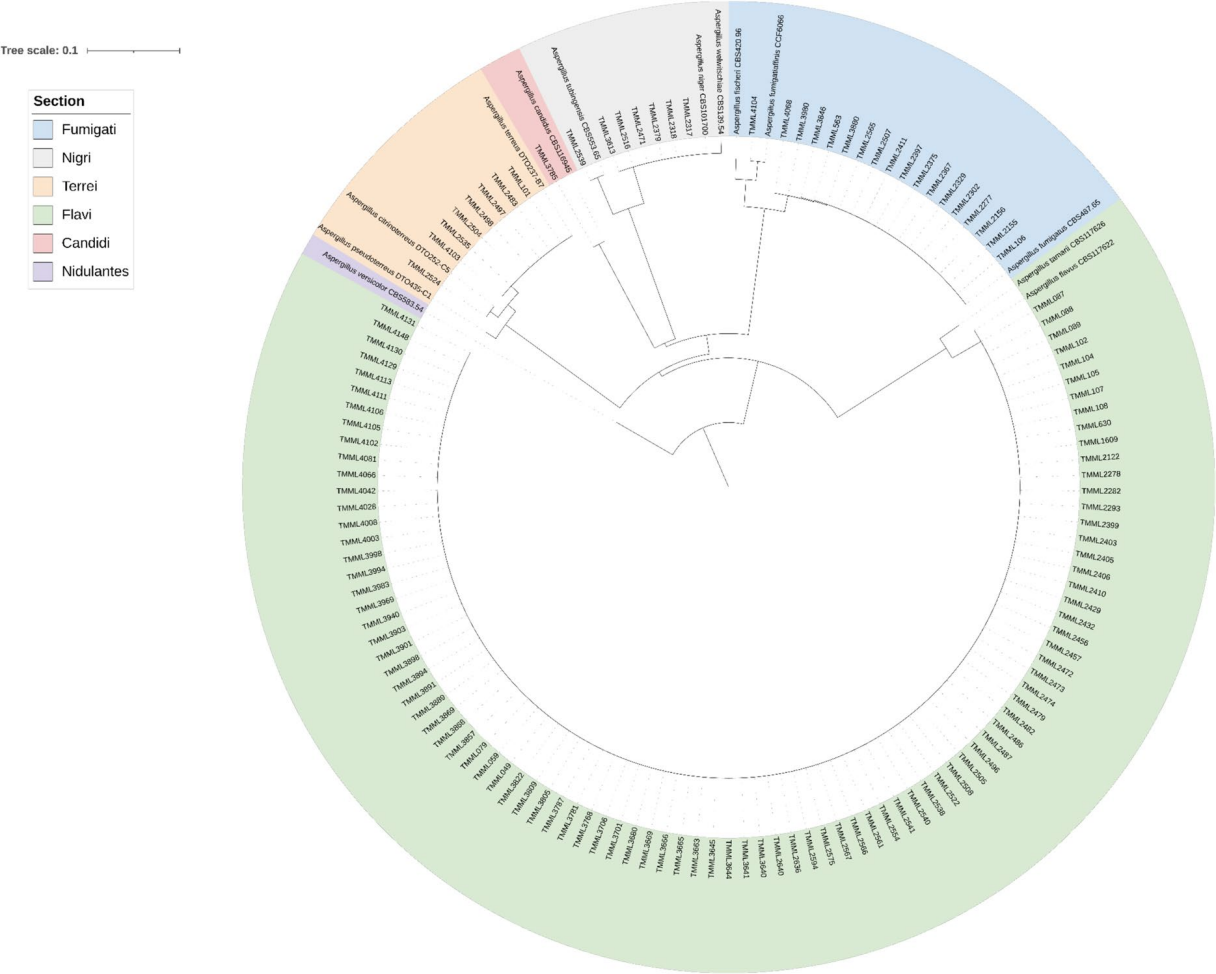
Phylogenetic tree based on alignment of calmodulin sequences of *Aspergillus* species. The tree was rooted to *A. versicolor* and isolates were colored after the corresponding section

*CaM* sequencing was performed for accurate species identification on all 127 *Aspergillus* isolates. Sequence comparisons revealed that among the 127 isolates, 73% (n = 93) belonged to *A. flavus *sensu stricto*,* 13% (n = 17) to *A. fumigatus *sensu stricto, 5% (n = 7) to *A. terreus *sensu stricto, 5% (n = 6) to *A. niger *sensu stricto*,* and 1% (n = 1) to *A. candidus, A. citrinoterreus*, *A. tubingensis* and *A. fumigatiaffinis* each, (Fig. 1). The latter three species belong to the sections *Terrei*, *Nigri* and *Fumigati*, respectively. For *A. flavus*, the portion of proven aspergillosis was higher when compared to *A. fumigatus*, *A. niger* and *A. terreus* (Fig. S1). For the three patients from whom two isolates were recovered, the same species was identified. The rare and cryptic *Aspergillus* species were all collected from patients with possible aspergillosis and the *A. citrinoterreus* and *A. tubingensis* were collected from BAL samples, while the source of the *A. fumigatiaffinis* could not be retrieved due to the retrospective nature of the study.

### Antifungal Resistance Investigation

In vitro AFST according to CLSI M38 guidelines was performed on 127 *Aspergillus* isolates. With application of available ECVs, no non-wild type isolates were found. MECs of micafungin and anidulafungin were low with either ≤ 0.008 µg/mL or 0.016 µg/mL for all tested strains (Table S3). For the triazoles, MICs of *A. flavus, A. fumigatus, A. niger* and *A. terreus* were comparable and the MIC_50_ mostly differed by one to two-fold dilutions between species (Table 1). Posaconazole displayed the highest in vitro activity with a MIC_50_ of 0.031–0.125 µg/mL, while voriconazole demonstrated the lowest in vitro activity with a MIC_50_ of 0.25–0.5 µg/mL for the four above mentioned species. Amphotericin B MICs of *A. flavus* were elevated in comparison to the other species, with its MIC_50_ being fourfold higher than *A. fumigatus*. *A. citrinoterreus* had an elevated amphotericin B MIC, while *A. fumigatiaffinis* also had elevated MICs against amphotericin B and itraconazole (Table S3).

**Table 1:** In vitro antifungal susceptibility testing metrics of *Aspergillus flavus *sensu stricto (n = 93), *Aspergillus fumigatus *sensu stricto (n = 17), *Aspergillus terreus *sensu stricto (n = 7) and *Aspergillus niger *sensu stricto (n = 6) according to CLSI M38 guidelines

Antifungal	Species	Range	Mode	GM	MIC_50_	MIC_90_
Amphotericin B	*A. flavus*	0.5–4	1	0.823	1	2
	*A. fumigatus*	0.125–0.25	0.25	0.230	0.25	0.25
	*A. niger*	0.063–0.125	0.125	0.112	0.125	NA
	*A. terreus*	0.25–1	0.25	0.371	0.25	NA
Voriconazole	*A. flavus*	0.25–1	1	0.635	0.5	1
	*A. fumigatus*	0.125–1	0.5	0.480	0.5	1
	*A. niger*	0.25–0.5	0.25	0.315	0.25	NA
	*A. terreus*	0.25–1	0.25	0.410	0.25	NA
Itraconazole	*A. flavus*	0.063–0.5	0.125	0.154	0.125	0.25
	*A. fumigatus*	0.063–0.5	0.25	0.196	0.25	0.25
	*A. niger*	0.125–0.5	0.25	0.250	0.25	NA
	*A. terreus*	0.031–0.25	0.125	0.138	0.125	NA
Posaconazole	*A. flavus*	0.031–0.125	0.063	0.068	0.063	0.125
	*A. fumigatus*	0.031–0.063	0.063	0.051	0.063	0.125
	*A. niger*	0.031–0.125	0.125	0.079	0.125	NA
	*A. terreus*	0.031–0.063	0.031	0.038	0.031	NA
Isavuconazole	*A. flavus*	0.25–1	0.5	0.555	0.5	1
	*A. fumigatus*	0.125–1	0.5	0.391	0.5	1
	*A. niger*	0.25–0.5	0.5	0.354	0.5	NA
	*A. terreus*	0.25–0.5	0.25	0.276	0.25	NA

All metrics in µg/mL

GM, geometric mean; MIC, minimum inhibitory concentration; NA, non-applicable

### STR Genotyping

**Fig. 2:**
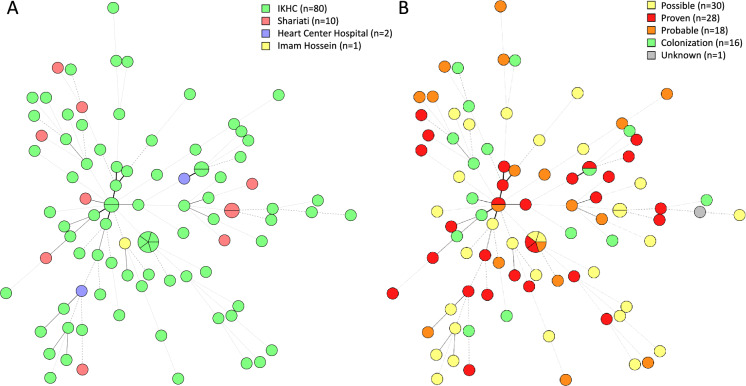
Cluster analysis of 93 *Aspergillus flavus* isolates based on short tandem repeat genotyping colored on hospital (**A**) and disease classification (**B**). Branch lengths of the minimum-spanning tree indicate similarity between isolates with thick solid lines (variation in one marker), thin solid lines (variation in two markers), thin dashed lines (variation in three markers) and thin dotted lines (variation in four or more markers). The number of isolates per classification is shown in the color key

**Fig. 3:**
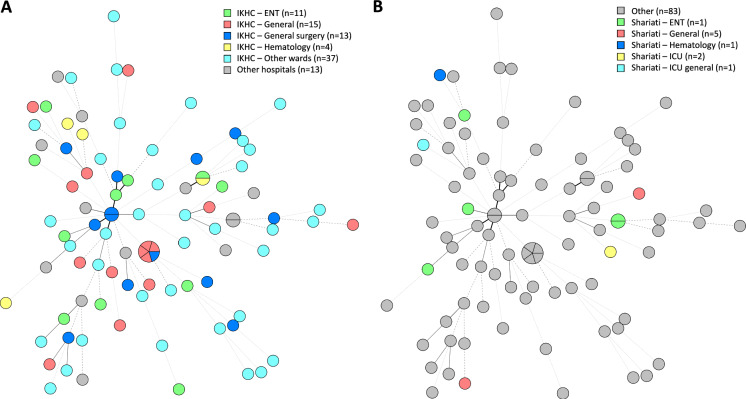
Cluster analysis of 93 *Aspergillus flavus* isolates based on short tandem repeat genotyping colored after hospital and corresponding wards for IKHC (**A**) and Shariati (**B**) hospital. Branch lengths indicate similarity between isolates, with thick solid lines (variation in one marker), thin solid lines (variation in two markers), thin dashed lines (variation in three markers) and thin dotted lines (variation in four or more markers)

For *A. flavus, A. fumigatus* and *A. terreus*, isolates were genotyped to investigate clonal transmission. Among the 93 *A. flavus* isolates, a total of 86 genotypes was found. Among these 86 genotypes, 82 were represented by a single isolate; three represented by two isolates each; and one by five isolates (Fig. 2A)*.* Each of the four clusters contained isolates from different patients from the same hospital with three out of four clusters containing at least one patient with proven aspergillosis (Fig. 2B). For three out of four clusters, including the largest comprising five patients, most isolates were allocated to the same ward and isolates were collected within five months from each other (Fig. 3**)**. Most other genotypes differed three or more STR markers from each other (Fig. S2). Two isolates from one patient with proven aspergillosis were nearly identical with one copy number difference in one marker, while in two otherisolates from a patient with possible aspergillosis, copy number variation was found for all nine markers.

**Fig. 4:**
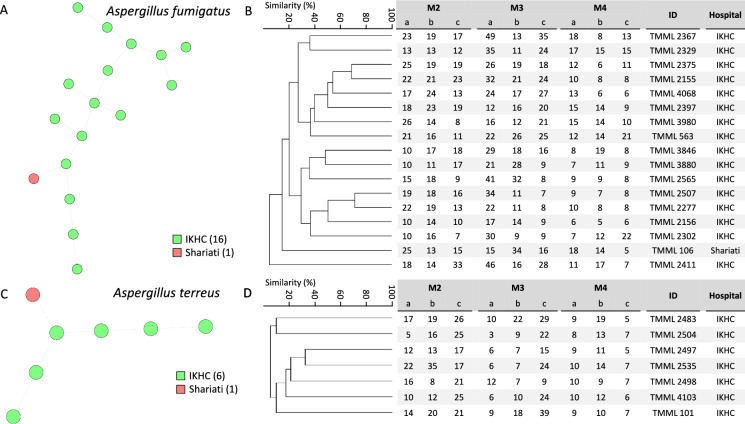
Cluster analysis of 17 *Aspergillus fumigatus* and 7 *Aspergillus terreus* isolates according to short tandem repeat genotyping. Branch lengths of the minimum-spanning tree indicate similarity between *A. fumigatus* isolates with thin dotted lines (variation in four or more markers (**A**). Isolates are colored after the hospital and the number is shown in the color key. Branch lengths of the UPGMA dendrograms indicate relatedness and copy numbers for each marker are shown for *A. fumigatus* (**B**) and *A. terreus* (**C**)

In contrast, all *A. fumigatus* and *A. terreus* isolates displayed unique genotypes and differed from each other in at least five markers (Fig. 4). For one patient with proven infection, two *A. fumigatus* isolates were collected and these differed in eight of out nine markers.

## Discussion

The current study reports 124 patients with isolated *Aspergillus* species ranging from colonization to proven aspergillosis from Iran. Molecular identification demonstrated *A. flavus *sensu stricto as the most common species. Previous studies on clinical *Aspergillus* samples from Iran and other sub- and tropical countries such as India also indicated *A. flavus* as the dominant species [16, 17], which is in contrast to European countries where *A. fumigatus* is most common [4]. In addition, *A. fumigatus *sensu stricto and *A. terreus *sensu stricto were frequently found, which are often involved in invasive aspergillosis as well [20]. Notable findings include one case of possible aspergillosis by *A. candidus* which is rarely implicated in human disease [18]. Other rarely found species were *A. citrinoterreus*, *A. tubingensis* and *A. fumigatiaffinis* which can only be identified with molecular techniques [19, 20]. Given that these cryptic species often display reduced susceptibility, accurate species identification is worthwhile in cases that do not respond to treatment, since these patients were diagnosed with possible aspergillosis [21].

With CLSI microbroth dilution, all isolates from species with available clinical breakpoints and ECVs were classified as susceptible to azoles and echinocandins [10, 11]. These finding are in line with previous studies on resistance investigation for clinical *Aspergillus* isolates from Iran, where resistance appeared to be rare and was mostly restricted to *A. fumigatus* [22]. Large Iranian clinical studies found overall azole resistance at a few percent, while other studies found no resistance for *A. fumigatus* [23, 24]. Triazoles remain therefore the best choice as first-line treatment for aspergillosis or prophylaxis, given that they are available [25]. *A. fumigatus* azole resistance rates for environmental isolates (9%), appeared to be higher than clinical isolates (4%) in Iran [26]. Most resistant isolates harbor the globally reported TR_34_/L98H mutation [27]. This clinical susceptibility contrasts with European countries such as the United Kingdom or the Netherlands where clinical resistance rates of *A. fumigatus* often exceeds 10% [26]. Extensive agricultural azole usage is known to induce resistance and coincides with emerging clinical resistance, as humans acquire these strains from the environment [27]. A Dutch study showed that fungicide-containing stockpiles of decaying plant waste act as hotspots, with roughly half of the recovered isolates showing resistance to itraconazole [28]. Surveillance in Iran is therefore warranted to monitor resistance in clinical isolates which is associated with increased mortality and clinical failure [29].

Elevated MICs were found in *A. flavus* for amphotericin B (0.5–4 µg/mL) in comparison to most other species (often ≤ 0.25 µg/mL) in the current study. *A. flavus* is known to exhibit variable amphotericin B MICs, and high MICs (≥ 2 µg/mL) correlate to clinical failure [16]. Another frequent aspergillosis causative agent is *A. terreus*, that is also known to display elevated amphotericin B MICs [30], although it was not found here. The single *A. citrinoterreus* isolate had an elevated amphotericin B MIC of 1 µg/mL, which has been reported earlier [31]. The small sample size of the current study might explain the absence of *A. terreus* strains with elevated MICs. Taken together, voriconazole remains the drug of choice to treat aspergillosis, which showed excellent in vitro activity against all strains in the current study.

STR genotyping of *A. flavus, A. fumigatus* and *A. terreus* demonstrated an overall high genetic diversity. Isolates of the latter two species displayed solely unique genotypes, which is in line with earlier studies showing wide genotypic variation [32]. Of note, the sample size of these two species was low, reducing the likelihood of detecting clusters. In contrast, identical genotypes were found for 11 *A. flavus* isolates grouped in four clusters of two to five isolates, indicating a close genetic relatedness. Whole genome sequencing (WGS) is required to confirm clonal relatedness [33]. Nonetheless, since most isolates in each of three clusters originated from the same ward and were collected within a relatively short time frame, these isolates likely originated from the same nosocomial source. A common source outside the hospital environment like a shared workspace or housing facility is less likely but cannot be excluded. This is one of the first studies suggesting nosocomial transmission of *A. flavus* [34]. To date, only a handful of studies reported *A. flavus* nosocomial clusters from Europe or the USA, making this study the first report from Asia. Construction work is often associated with *Aspergillus* outbreaks as spores can accumulate, for example in air condition filters, and subsequently be released during renovation or demolition activities [34]. Air filtration within the hospital environment could aid in reducing the spore count to subsequently prevent healthcare-associated outbreaks [35]. In addition, implementation of robust environmental control measures accompanied by active infection surveillance systems are essential to enable the detection of potential outbreak scenarios.

A limitation of this study is that besides *A. flavus*, *A. fumigatus* and *A. terreus*, other species were not genotyped, which makes potentially missing nosocomial transmission by these species. Another limitation is the absence of genotyped *A. flavus* from the environment like air filters in hospitals but also from workplaces and homes. Consequently, the source of the *A. flavus* clusters remains unknown.

To conclude, aspergillosis or colonization cases, *A. flavus* was the most common species, followed by *A. fumigatus* and *A. terreus*. Rare species include *A. candidus* and *C. fumigatiaffinis*. *Aspergillus* isolates were found to be completely susceptible or wild type, although cryptic species displayed reduced susceptibility. With genotyping on *Aspergillus* isolates, an overall high genetic diversity was found. Outstanding were three *A. flavus* clusters that each consisted of patients admitted to the same wards within several months in time. This strongly indicates nosocomial transmission events of *A. flavus*, highlighting the need for air filtration and stricter infection prevention practices, although WGS is needed for decisive confirmation.

## Supplementary Information

Below is the link to the electronic supplementary material.

Supplementary file1 (PPTX 55 KB)

Supplementary file2 (PPTX 834 KB)

Supplementary file3 (DOCX 152 KB)
