# Supplementary material for: Genotypic and Phenotypic Investigation of Clinical Aspergillus isolates from Iran Indicates Nosocomial Transmission Events of Aspergillus flavus
Source: Mycopathologia. 2025 Aug 30;190(5):79. doi: 10.1007/s11046-025-00988-w (PMC12398430; doi:10.1007/s11046-025-00988-w)
Supplement: Supplementary file 1 — Supplementary file1 (PPTX 55 KB) [file 11046_2025_988_MOESM1_ESM.pptx]

## Slide 1
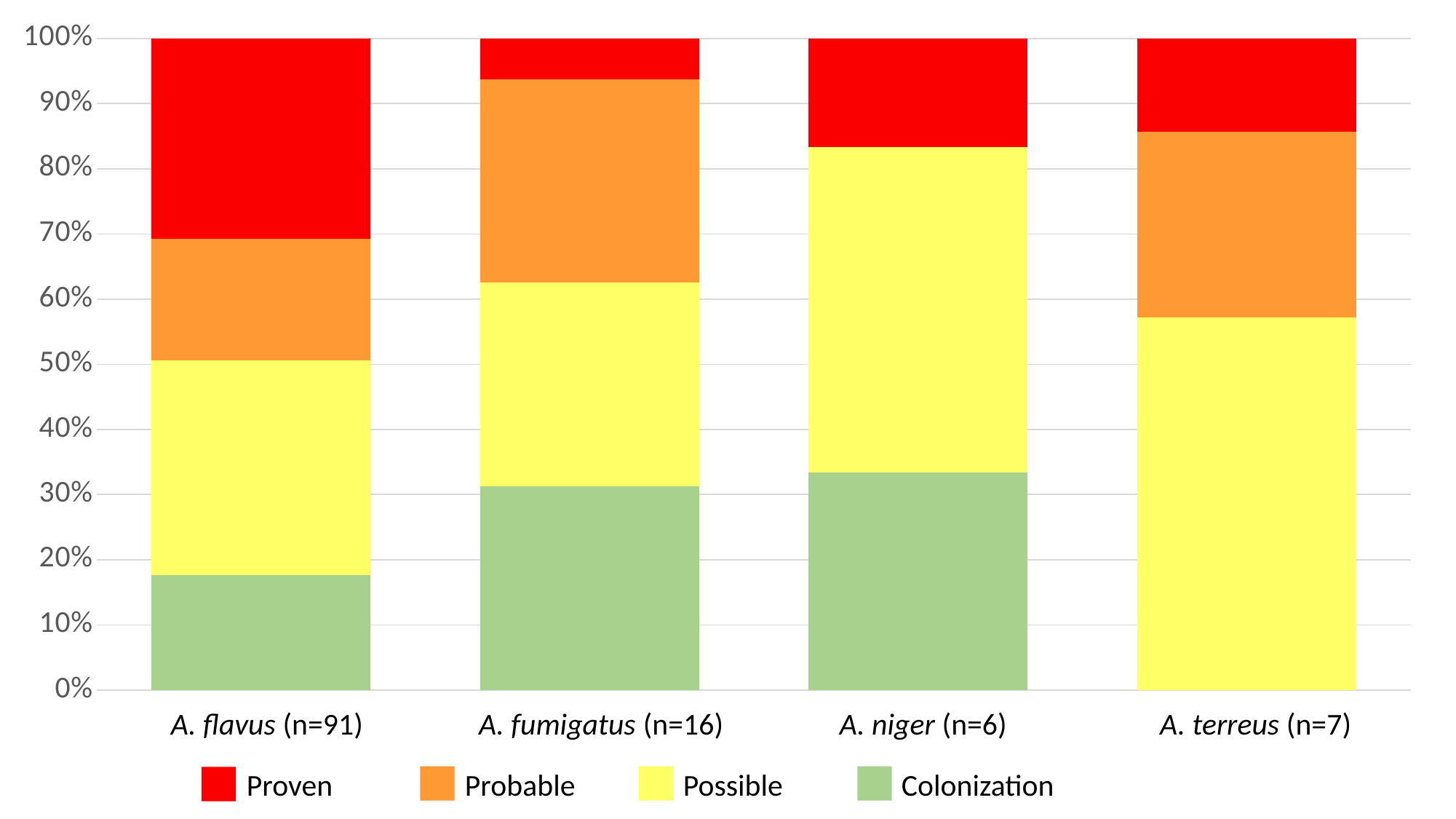

### Chart
| Category | | | | |
|---|---|---|---|---|
| A. flavus | 16.0 | 30.0 | 17.0 | 28.0 |
| A. fumigatus | 5.0 | 5.0 | 5.0 | 1.0 |
| A. niger | 2.0 | 3.0 | None | 1.0 |
| A. terreus | None | 4.0 | 2.0 | 1.0 |A. flavus (n=91)	 A. fumigatus (n=16)	 A. niger (n=6)		 A. terreus (n=7)
Proven		Probable	Possible		Colonization
