# Supplementary material for: Genotypic and Phenotypic Investigation of Clinical Aspergillus isolates from Iran Indicates Nosocomial Transmission Events of Aspergillus flavus
Source: Mycopathologia. 2025 Aug 30;190(5):79. doi: 10.1007/s11046-025-00988-w (PMC12398430; doi:10.1007/s11046-025-00988-w)
Supplement: Supplementary file 2 — Supplementary file2 (PPTX 834 KB) [file 11046_2025_988_MOESM2_ESM.pptx]

## Slide 1
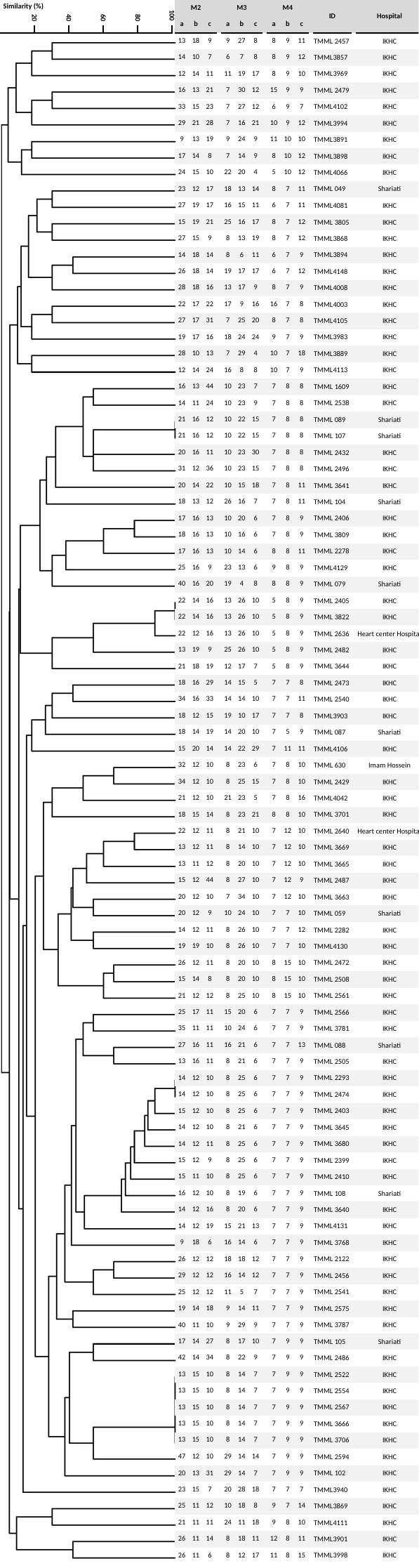

Similarity (%)
| M2 | | | | M3 | | | | M4 | | | | ID | | Hospital |
| --- | --- | --- | --- | --- | --- | --- | --- | --- | --- | --- | --- | --- | --- | --- |
| a | b | c | | a | b | c | | a | b | c | | | | |
| 13 | 18 | 9 | | 9 | 27 | 8 | | 8 | 9 | 11 | | TMML 2457 | | IKHC |
| 14 | 10 | 7 | | 6 | 7 | 8 | | 8 | 9 | 12 | | TMML3857 | | IKHC |
| 12 | 14 | 11 | | 11 | 19 | 17 | | 8 | 9 | 10 | | TMML3969 | | IKHC |
| 16 | 13 | 21 | | 7 | 30 | 12 | | 15 | 9 | 9 | | TMML 2479 | | IKHC |
| 33 | 15 | 23 | | 7 | 27 | 12 | | 6 | 9 | 7 | | TMML4102 | | IKHC |
| 29 | 21 | 28 | | 7 | 16 | 21 | | 10 | 9 | 12 | | TMML3994 | | IKHC |
| 9 | 13 | 19 | | 9 | 24 | 9 | | 11 | 10 | 10 | | TMML3891 | | IKHC |
| 17 | 14 | 8 | | 7 | 14 | 9 | | 8 | 10 | 12 | | TMML3898 | | IKHC |
| 24 | 15 | 10 | | 22 | 20 | 4 | | 5 | 10 | 12 | | TMML4066 | | IKHC |
| 23 | 12 | 17 | | 18 | 13 | 14 | | 8 | 7 | 11 | | TMML 049 | | Shariati |
| 27 | 19 | 17 | | 16 | 15 | 11 | | 6 | 7 | 11 | | TMML4081 | | IKHC |
| 15 | 19 | 21 | | 25 | 16 | 17 | | 8 | 7 | 12 | | TMML 3805 | | IKHC |
| 27 | 15 | 9 | | 8 | 13 | 19 | | 8 | 7 | 12 | | TMML3868 | | IKHC |
| 14 | 18 | 14 | | 8 | 6 | 11 | | 6 | 7 | 9 | | TMML3894 | | IKHC |
| 26 | 18 | 14 | | 19 | 17 | 17 | | 6 | 7 | 12 | | TMML4148 | | IKHC |
| 28 | 18 | 16 | | 13 | 17 | 9 | | 8 | 7 | 9 | | TMML4008 | | IKHC |
| 22 | 17 | 22 | | 17 | 9 | 16 | | 16 | 7 | 8 | | TMML4003 | | IKHC |
| 27 | 17 | 31 | | 7 | 25 | 20 | | 8 | 7 | 8 | | TMML4105 | | IKHC |
| 19 | 17 | 16 | | 18 | 24 | 24 | | 9 | 7 | 9 | | TMML3983 | | IKHC |
| 28 | 10 | 13 | | 7 | 29 | 4 | | 10 | 7 | 18 | | TMML3889 | | IKHC |
| 12 | 14 | 24 | | 16 | 8 | 8 | | 10 | 7 | 9 | | TMML4113 | | IKHC |
| 16 | 13 | 44 | | 10 | 23 | 7 | | 7 | 8 | 8 | | TMML 1609 | | IKHC |
| 14 | 11 | 24 | | 10 | 23 | 9 | | 7 | 8 | 8 | | TMML 2538 | | IKHC |
| 21 | 16 | 12 | | 10 | 22 | 15 | | 7 | 8 | 8 | | TMML 089 | | Shariati |
| 21 | 16 | 12 | | 10 | 22 | 15 | | 7 | 8 | 8 | | TMML 107 | | Shariati |
| 20 | 16 | 11 | | 10 | 23 | 30 | | 7 | 8 | 8 | | TMML 2432 | | IKHC |
| 31 | 12 | 36 | | 10 | 23 | 15 | | 7 | 8 | 8 | | TMML 2496 | | IKHC |
| 20 | 14 | 22 | | 10 | 15 | 18 | | 7 | 8 | 11 | | TMML 3641 | | IKHC |
| 18 | 13 | 12 | | 26 | 16 | 7 | | 7 | 8 | 11 | | TMML 104 | | Shariati |
| 17 | 16 | 13 | | 10 | 20 | 6 | | 7 | 8 | 9 | | TMML 2406 | | IKHC |
| 18 | 16 | 13 | | 10 | 16 | 6 | | 7 | 8 | 9 | | TMML 3809 | | IKHC |
| 17 | 16 | 13 | | 10 | 14 | 6 | | 8 | 8 | 11 | | TMML 2278 | | IKHC |
| 25 | 16 | 9 | | 23 | 13 | 6 | | 9 | 8 | 9 | | TMML4129 | | IKHC |
| 40 | 16 | 20 | | 19 | 4 | 8 | | 8 | 8 | 9 | | TMML 079 | | Shariati |
| 22 | 14 | 16 | | 13 | 26 | 10 | | 5 | 8 | 9 | | TMML 2405 | | IKHC |
| 22 | 14 | 16 | | 13 | 26 | 10 | | 5 | 8 | 9 | | TMML 3822 | | IKHC |
| 22 | 12 | 16 | | 13 | 26 | 10 | | 5 | 8 | 9 | | TMML 2636 | | Heart center Hospital |
| 13 | 19 | 9 | | 25 | 26 | 10 | | 5 | 8 | 9 | | TMML 2482 | | IKHC |
| 21 | 18 | 19 | | 12 | 17 | 7 | | 5 | 8 | 9 | | TMML 3644 | | IKHC |
| 18 | 16 | 29 | | 14 | 15 | 5 | | 7 | 7 | 8 | | TMML 2473 | | IKHC |
| 34 | 16 | 33 | | 14 | 14 | 10 | | 7 | 7 | 11 | | TMML 2540 | | IKHC |
| 18 | 12 | 15 | | 19 | 10 | 17 | | 7 | 7 | 8 | | TMML3903 | | IKHC |
| 18 | 14 | 19 | | 14 | 20 | 10 | | 7 | 5 | 9 | | TMML 087 | | Shariati |
| 15 | 20 | 14 | | 14 | 22 | 29 | | 7 | 11 | 11 | | TMML4106 | | IKHC |
| 32 | 12 | 10 | | 8 | 23 | 6 | | 7 | 8 | 10 | | TMML 630 | | Imam Hossein |
| 34 | 12 | 10 | | 8 | 25 | 15 | | 7 | 8 | 10 | | TMML 2429 | | IKHC |
| 21 | 12 | 10 | | 21 | 23 | 5 | | 7 | 8 | 16 | | TMML4042 | | IKHC |
| 18 | 15 | 14 | | 8 | 23 | 21 | | 8 | 8 | 10 | | TMML 3701 | | IKHC |
| 22 | 12 | 11 | | 8 | 21 | 10 | | 7 | 12 | 10 | | TMML 2640 | | Heart center Hospital |
| 13 | 12 | 11 | | 8 | 14 | 10 | | 7 | 12 | 10 | | TMML 3669 | | IKHC |
| 13 | 11 | 12 | | 8 | 20 | 10 | | 7 | 12 | 10 | | TMML 3665 | | IKHC |
| 15 | 12 | 44 | | 8 | 27 | 10 | | 7 | 12 | 9 | | TMML 2487 | | IKHC |
| 20 | 12 | 10 | | 7 | 34 | 10 | | 7 | 12 | 10 | | TMML 3663 | | IKHC |
| 20 | 12 | 9 | | 10 | 24 | 10 | | 7 | 7 | 10 | | TMML 059 | | Shariati |
| 14 | 12 | 11 | | 8 | 26 | 10 | | 7 | 7 | 12 | | TMML 2282 | | IKHC |
| 19 | 19 | 10 | | 8 | 26 | 10 | | 7 | 7 | 10 | | TMML4130 | | IKHC |
| 26 | 12 | 11 | | 8 | 20 | 10 | | 8 | 15 | 10 | | TMML 2472 | | IKHC |
| 15 | 14 | 8 | | 8 | 20 | 10 | | 8 | 15 | 10 | | TMML 2508 | | IKHC |
| 21 | 12 | 12 | | 8 | 25 | 10 | | 8 | 15 | 10 | | TMML 2561 | | IKHC |
| 25 | 17 | 11 | | 15 | 20 | 6 | | 7 | 7 | 9 | | TMML 2566 | | IKHC |
| 35 | 11 | 11 | | 10 | 24 | 6 | | 7 | 7 | 9 | | TMML 3781 | | IKHC |
| 27 | 16 | 11 | | 16 | 21 | 6 | | 7 | 7 | 13 | | TMML 088 | | Shariati |
| 13 | 16 | 11 | | 8 | 21 | 6 | | 7 | 7 | 9 | | TMML 2505 | | IKHC |
| 14 | 12 | 10 | | 8 | 25 | 6 | | 7 | 7 | 9 | | TMML 2293 | | IKHC |
| 14 | 12 | 10 | | 8 | 25 | 6 | | 7 | 7 | 9 | | TMML 2474 | | IKHC |
| 15 | 12 | 10 | | 8 | 25 | 6 | | 7 | 7 | 9 | | TMML 2403 | | IKHC |
| 14 | 12 | 10 | | 8 | 21 | 6 | | 7 | 7 | 9 | | TMML 3645 | | IKHC |
| 14 | 12 | 11 | | 8 | 25 | 6 | | 7 | 7 | 9 | | TMML 3680 | | IKHC |
| 15 | 12 | 9 | | 8 | 25 | 6 | | 7 | 7 | 9 | | TMML 2399 | | IKHC |
| 15 | 11 | 10 | | 8 | 25 | 6 | | 7 | 7 | 9 | | TMML 2410 | | IKHC |
| 16 | 12 | 10 | | 8 | 19 | 6 | | 7 | 7 | 9 | | TMML 108 | | Shariati |
| 14 | 12 | 16 | | 8 | 20 | 6 | | 7 | 7 | 9 | | TMML 3640 | | IKHC |
| 14 | 12 | 19 | | 15 | 21 | 13 | | 7 | 7 | 9 | | TMML4131 | | IKHC |
| 9 | 18 | 6 | | 16 | 14 | 6 | | 7 | 7 | 9 | | TMML 3768 | | IKHC |
| 26 | 12 | 12 | | 18 | 18 | 12 | | 7 | 7 | 9 | | TMML 2122 | | IKHC |
| 29 | 12 | 12 | | 16 | 14 | 12 | | 7 | 7 | 9 | | TMML 2456 | | IKHC |
| 25 | 12 | 12 | | 11 | 5 | 7 | | 7 | 7 | 9 | | TMML 2541 | | IKHC |
| 19 | 14 | 18 | | 9 | 14 | 11 | | 7 | 7 | 9 | | TMML 2575 | | IKHC |
| 40 | 11 | 10 | | 9 | 29 | 9 | | 7 | 7 | 9 | | TMML 3787 | | IKHC |
| 17 | 14 | 27 | | 8 | 17 | 10 | | 7 | 9 | 9 | | TMML 105 | | Shariati |
| 42 | 14 | 34 | | 8 | 22 | 9 | | 7 | 9 | 9 | | TMML 2486 | | IKHC |
| 13 | 15 | 10 | | 8 | 14 | 7 | | 7 | 9 | 9 | | TMML 2522 | | IKHC |
| 13 | 15 | 10 | | 8 | 14 | 7 | | 7 | 9 | 9 | | TMML 2554 | | IKHC |
| 13 | 15 | 10 | | 8 | 14 | 7 | | 7 | 9 | 9 | | TMML 2567 | | IKHC |
| 13 | 15 | 10 | | 8 | 14 | 7 | | 7 | 9 | 9 | | TMML 3666 | | IKHC |
| 13 | 15 | 10 | | 8 | 14 | 7 | | 7 | 9 | 9 | | TMML 3706 | | IKHC |
| 47 | 12 | 10 | | 29 | 14 | 14 | | 7 | 9 | 9 | | TMML 2594 | | IKHC |
| 20 | 13 | 31 | | 29 | 14 | 7 | | 7 | 9 | 9 | | TMML 102 | | IKHC |
| 23 | 15 | 7 | | 20 | 28 | 18 | | 7 | 7 | 7 | | TMML3940 | | IKHC |
| 25 | 11 | 12 | | 10 | 18 | 8 | | 9 | 7 | 14 | | TMML3869 | | IKHC |
| 21 | 11 | 11 | | 24 | 11 | 18 | | 9 | 8 | 10 | | TMML4111 | | IKHC |
| 26 | 11 | 14 | | 8 | 18 | 11 | | 12 | 8 | 11 | | TMML3901 | | IKHC |
| 26 | 11 | 6 | | 8 | 12 | 17 | | 11 | 8 | 15 | | TMML3998 | | IKHC |
100
20
40
60
80
