# Supplementary material for: Genotypic and Phenotypic Investigation of Clinical Aspergillus isolates from Iran Indicates Nosocomial Transmission Events of Aspergillus flavus
Source: Mycopathologia. 2025 Aug 30;190(5):79. doi: 10.1007/s11046-025-00988-w (PMC12398430; doi:10.1007/s11046-025-00988-w)
Supplement: Supplementary file 3 — Supplementary file3 (DOCX 152 KB) [file 11046_2025_988_MOESM3_ESM.docx]

**Figure S1: Number of patients with aspergillosis diagnosis according to EORTC/MSGERC criteria for *A. flavus*, *A. fumigatus*, *A. niger* and *A. terreus*.**

**Figure S2: Dendrogram of 93 *Aspergillus flavus* isolates based on short tandem repeat genotyping.** Lengths of the UPGMA dendrogram indicate relatedness and copy numbers for each marker are shown.

**Supplementary**

**Supplementary table 1: Calmodulin (*CaM*) reference sequences from *Aspergillus* strains with accession numbers.** All sequences were extracted from the National Center for Biotechnology (NCBI) nucleotide database.

| **ID** | **Species** | **Section** | **Accession number** |
| --- | --- | --- | --- |
| CCF 6066 | *A. fumigatiaffinis* | *Fumigati* | LS999592.1 |
| CBS 487.65 | *A. fumigatus* | *Fumigati* | AB259965.1 |
| CBS 420.96 | *A. fischeri* | *Fumigati* | AY870688.1 |
| CBS 117622 | *A. flavus* | *Flavi* | EF202068.1 |
| CBS 117626 | *A. tamarii* | *Flavi* | EF202033.1 |
| CBS 101700 | *A. niger* | *Nigri* | GU195633.1 |
| CBS 139.54 | *A. welwitschiae* | *Nigri* | KC480196.1 |
| CBS 553.65 | *A. tubingensis* | *Nigri* | OP081904.1 |
| DTO 237-B7 | *A. terreus* | *Terrei* | KJ775299.1 |
| DTO 252-C5 | *A. citrinoterreus* | *Terrei* | MG490539.1 |
| DTO 435-C1 | *A. pseudoterreus* | *Terrei* | MW671051.1 |
| CBS 116945 | *A. candidus* | *Candidi* | EU076309.1 |
| CBS 583.54 | *A. versicolor* | *Nidulantes* | FN594604.1 |

**Supplementary table 2: Clinical information on all 127 *Aspergillus* isolates included in the current study.**

| **ID** | **Morphology** | **Molecular ID** | **Source** | **Hospital** | **Sex** | **Ward** | **Years** | **Diagnosis** |
| --- | --- | --- | --- | --- | --- | --- | --- | --- |
| TMML 101 | *A. terreus* | *A. terreus* | BAL | 2 | F | General | 2020 | Possible |
| TMML 102 | *A. flavus* | *A. flavus* | Sinus | 2 | F | ENT | 2020 | Proven |
| TMML 104 | *A. flavus* | *A. flavus* | Tracheal | 1 | M | ICU | 2020 | Possible |
| TMML 105 | *A. flavus* | *A. flavus* | Tracheal | 1 | M | General | 2020 | Colonization |
| TMML 106 | *A. fumigatus* | *A. fumigatus* | Tracheal | 1 | F | General | 2020 | Colonization |
| TMML 107 | *A. flavus* | *A. flavus* | Nose | 1 | M | General | 2020 | Possible |
| TMML 108 | *A. flavus* | *A. flavus* | Sputum | 1 | F | Respiratory unit | 2020 | Colonization |
| TMML 1609 | *A. flavus* | *A. flavus* | BAL | 2 | NA | NA | NA | NA |
| TMML 2122 | *A. flavus* | *A. flavus* | Sputum | 2 | M | General | 2021 | Colonization |
| TMML 2155 | *A. fumigatus* | *A. fumigatus* | Sputum | 2 | F | General | 2021 | Colonization |
| TMML 2156 | *A. fumigatus* | *A. fumigatus* | Sputum | 2 | M | ICU | 2021 | Colonization |
| TMML 2277 | *A. fumigatus* | *A. fumigatus* | Tracheal | 2 | M | ICU general | 2021 | Probable |
| TMML 2278 | *A. flavus* | *A. flavus* | Sputum | 2 | F | ID | 2021 | Probable |
| TMML 2282 | *A. flavus* | *A. flavus* | Right Nose | 2 | F | Surgery | 2021 | Proven |
| TMML 2293 | *A. flavus* | *A. flavus* | Sputum | 2 | M | General (surgical) | 2021 | Probable |
| TMML 2302 | *A. fumigatus* | *A. fumigatus* | Sputum | 2 | F | Women Heart | 2021 | Colonization |
| TMML 2317 | *A. niger* | *A. niger* | Sputum | 2 | NA | NA | NA | NA |
| TMML 2318 | *A. niger* | *A. niger* | Sputum | 2 | F | ICU | 2021 | Possible |
| TMML 2329 | *A. fumigatus* | *A. fumigatus* | Tracheal | 2 | M | General (surgical) | 2021 | Possible |
| TMML 2367 | *A. fumigatus* | *A. fumigatus* | Tracheal | 2 | M | General | 2021 | Possible |
| TMML 2375 | *A. fumigatus* | *A. fumigatus* | Tracheal | 2 | F | ICU Lung | 2021 | Possible |
| TMML 2379 | *A. niger* | *A. niger* | Tracheal | 2 | NA | NA | NA | NA |
| TMML 2397 | *A. fumigatus* | *A. fumigatus* | Sputum | 2 | F | Hematology | 2021 | Probable |
| TMML 2399 | *A. flavus* | *A. flavus* | Right eye | 2 | M | General (surgical) | 2021 | Proven |
| TMML 2403 | *A. flavus* | *A. flavus* | Right turbinate | 2 | F | ENT | 2021 | Proven |
| TMML 2405 | *A. flavus* | *A. flavus* | Nose | 2 | M | ENT | 2021 | Proven |
| TMML 2406 | *A. flavus* | *A. flavus* | Lavage | 2 | M | Surgery | 2021 | Probable |
| TMML 2410 | *A. flavus* | *A. flavus* | Middle turbinate | 2 | F | ENT | 2021 | Probable |
| TMML 2411 | *A. fumigatus* | *A. fumigatus* | Sputum | 2 | F | Hematology | 2021 | Proven |
| TMML 2429 | *A. flavus* | *A. flavus* | Sputum | 2 | M | General (surgical) | 2021 | Proven |
| TMML 2432 | *A. flavus* | *A. flavus* | Wound | 2 | M | General (surgical) | 2021 | Proven |
| TMML 2456 | *A. flavus* | *A. flavus* | Sputum | 2 | F | General (surgical) | 2021 | Colonization |
| TMML 2457 | *A. flavus* | *A. flavus* | Nose | 2 | F | General (surgical) | 2021 | Possible |
| TMML 2471 | *A. niger* | *A. niger* | Right shoulder | 2 | F | ID | 2021 | Proven |
| TMML 2472 | *A. flavus* | *A. flavus* | Lavage | 2 | M | General (surgical) | 2021 | Possible |
| TMML 2473 | *A. flavus* | *A. flavus* | Nose | 2 | F | General (surgical) | 2021 | Possible |
| TMML 2474 | *A. flavus* | *A. flavus* | sinus- maxilla | 2 | M | General (surgical) | 2021 | Proven |
| TMML 2479 | *A. flavus* | *A. flavus* | Sinus | 2 | M | ID | 2021 | Proven |
| TMML 2482 | *A. flavus* | *A. flavus* | left orbit | 2 | M | ICU | 2021 | Proven |
| TMML 2483 | *A. restrictus* | *A. terreus* | Lavage | 2 | F | Neurology | 2021 | Probable |
| TMML 2486 | *A. flavus* | *A. flavus* | Nose | 2 | M | NA | 2021 | Colonization |
| TMML 2487 | *A. flavus* | *A. flavus* | Lavage | 2 | M | Heart surgery | 2022 | Colonization |
| TMML 2496 | *A. flavus* | *A. flavus* | Maxilla | 2 | F | ID | 2022 | Proven |
| TMML 2497 | *Aspergillus sp.* | *A. terreus* | Sinus | 2 | F | General (surgical) | 2022 | Proven |
| TMML 2498 | *Aspergillus sp.* | *A. terreus* | Sputum | 2 | M | Surgery | 2022 | Possible |
| TMML 2504 | *A. terreus* | *A. terreus* | Lavage | 2 | M | ICU | 2022 | Possible |
| TMML 2505 | *Aspergillus sp.* | *A. flavus* | Sputum | 2 | F | ICU | 2022 | Colonization |
| TMML 2507 | *A. fumigatus* | *A. fumigatus* | Sputum | 2 | NA | NA | NA | NA |
| TMML 2508 | *A. flavus* | *A. flavus* | Abscess | 2 | M | ID | 2022 | Possible |
| TMML 2516 | *A. niger* | *A. niger* | Trachea | 2 | M | General | 2022 | Possible |
| TMML 2522 | *A. flavus* | *A. flavus* | Brain Tissue | 2 | M | General | 2022 | Proven |
| TMML 2524 | *Aspergillus sp.* | *A. citrinoterreus* | Lavage | 2 | F | Neurology | 2021 | Possible |
| TMML 2535 | *A. terreus* | *A. terreus* | Tracheal | 2 | M | General | NA | Possible |
| TMML 2538 | *A. flavus* | *A. flavus* | Lavage | 2 | F | General | NA | Possible |
| TMML 2539 | *A. niger* | *A. tubingensis* | Lavage | 2 | F | Heart | NA | Possible |
| TMML 2540 | *A. flavus* | *A. flavus* | BAL | 2 | M | General (surgical) | NA | Probable |
| TMML 2541 | *A. flavus* | *A. flavus* | Sputum | 2 | M | Hematology | 2022 | Possible |
| TMML 2554 | *A. flavus* | *A. flavus* | BAL | 2 | M | General | 2022 | Proven |
| TMML 2561 | *A. flavus* | *A. flavus* | BAL | 2 | M | General | 2022 | Possible |
| TMML 2565 | *A. fumigatus* | *A. fumigatus* | Lavage | 2 | F | General | 2022 | Possible |
| TMML 2566 | *A. flavus* | *A. flavus* | Nasal Contents | 2 | F | General | 2022 | Possible |
| TMML 2567 | *A. flavus* | *A. flavus* | Nasal Contents | 2 | F | General (surgical) | 2022 | Possible |
| TMML 2575 | *A. flavus* | *A. flavus* | BAL | 2 | M | ENT | 2022 | Probable |
| TMML 2594 | *A. flavus* | *A. flavus* | BAL | 2 | M | Surgery | 2022 | Probable |
| TMML 2636 | *A. flavus* | *A. flavus* | Heart Tissue | 4 | M | NA | NA | Proven |
| TMML 2640 | *A. flavus* | *A. flavus* | Heart Tissue | 4 | M | NA | NA | Proven |
| TMML 3613 | *A. niger* | *A. niger* | BAL | 2 | M | General (surgical) | NA | Possible |
| TMML 3640 | *A. flavus* | *A. flavus* | Tissue | 2 | M | ID | 2022 | Proven |
| TMML 3641 | *A. flavus* | *A. flavus* | Ulcer | 2 | M | Emergency | 2022 | Colonization |
| TMML 3644 | *A. flavus* | *A. flavus* | Tracheal | 2 | M | ICU | 2022 | Colonization |
| TMML 3645 | *A. flavus* | *A. flavus* | Lavage | 2 | M | General (surgical) | 2022 | Colonization |
| TMML 3663 | *A. flavus* | *A. flavus* | Lavage | 2 | M | Kidney | 2022 | Colonization |
| TMML 3665 | *A. flavus* | *A. flavus* | BAL | 2 | M | ICU | 2022 | Probable |
| TMML 3666 | *A. flavus* | *A. flavus* | Sputum | 2 | F | General | 2022 | Possible |
| TMML 3669 | *A. flavus* | *A. flavus* | Abccess | 2 | F | ENT | 2022 | Possible |
| TMML 3680 | *A. flavus* | *A. flavus* | Lavage | 2 | M | ID | 2022 | Possible |
| TMML 3701 | *A. flavus* | *A. flavus* | Cornea | 2 | M | ID | 2022 | Proven |
| TMML 3706 | *A. flavus* | *A. flavus* | Nasal Contents | 2 | M | General | 2022 | Probable |
| TMML 3768 | *A. flavus* | *A. flavus* | BAL | 2 | F | Surgery | 2022 | Proven |
| TMML 3781 | *A. flavus* | *A. flavus* | BAL | 2 | F | General | 2022 | Probable |
| TMML 3785 | *A. candidus* | *A. candidus* | Lavage | 2 | M | Hematology | 2023 | Possible |
| TMML 3787 | *A. flavus* | *A. flavus* | Nasal Contents | 2 | F | ICU | 2023 | Possible |
| TMML 3805 | *A. flavus* | *A. flavus* | Nasal Contents | 2 | M | General | 2023 | Colonization |
| TMML 3809 | *A. flavus* | *A. flavus* | Sinus tissue | 2 | F | General | 2023 | Proven |
| TMML 3822 | *A. flavus* | *A. flavus* | Nasal Contents | 2 | M | Hematology | 2023 | Colonization |
| TMML 3846 | *A. fumigatus* | *A. fumigatus* | NA | 1 | F | General | 2023 | Probable |
| TMML 3857 | *A. flavus* | *A. flavus* | NA | 2 | F | ID | 2023 | Possible |
| TMML 3868 | *A. flavus* | *A. flavus* | NA | 2 | F | ICU | 2023 | Possible |
| TMML 3869 | *A. flavus* | *A. flavus* | NA | 2 | M | Hematology | 2023 | Possible |
| TMML 3880 | *A. fumigatus* | *A. fumigatus* | BAL | 2 | M | General | 2023 | Probable |
| TMML 3889 | *A. flavus* | *A. flavus* | NA | 2 | F | ID | 2023 | Possible |
| TMML 3891 | *A. flavus* | *A. flavus* | NA | 2 | M | Neurology | 2023 | Possible |
| TMML 3894 | *A. flavus* | *A. flavus* | NA | 1 | M | General | 2023 | Possible |
| TMML 3898 | *A. flavus* | *A. flavus* | NA | 2 | M | Hematology | 2023 | Possible |
| TMML 3901 | *A. flavus* | *A. flavus* | NA | 2 | F | Pediatric | 2023 | Possible |
| TMML 3903 | *A. flavus* | *A. flavus* | NA | 1 | M | ID | 2023 | Probable |
| TMML 3940 | *A. flavus* | *A. flavus* | NA | 2 | M | Pediatric | 2023 | Possible |
| TMML 3969 | *A. flavus* | *A. flavus* | NA | 2 | F | ICU | 2023 | Possible |
| TMML 3980 | *A. fumigatus* | *A. fumigatus* | NA | 1 | F | ICU | 2023 | Probable |
| TMML 3983 | *A. flavus* | *A. flavus* | NA | 2 | M | Urology | 2023 | Colonization |
| TMML 3994 | *A. flavus* | *A. flavus* | NA | 2 | M | ICU | 2023 | Probable |
| TMML 3998 | *A. flavus* | *A. flavus* | NA | 2 | F | ENT | 2023 | Probable |
| TMML 4003 | *A. flavus* | *A. flavus* | NA | 2 | F | Surgery | 2023 | Probable |
| TMML 4008 | *A. flavus* | *A. flavus* | NA | 2 | F | Heart | 2023 | Proven |
| TMML 4042 | *A. flavus* | *A. flavus* | NA | 2 | M | General | 2023 | Possible |
| TMML 4066 | *A. flavus* | *A. flavus* | NA | 1 | M | General (surgical) | NA | Possible |
| TMML 4068 | *A. fumigatus* | *A. fumigatus* | NA | 1 | F | ID | NA | Possible |
| TMML 4081 | *A. flavus* | *A. flavus* | NA | 2 | F | Hematology | 2023 | Proven |
| TMML 4102 | *A. flavus* | *A. flavus* | NA | 2 | F | Rheumatology | 2023 | Possible |
| TMML 4103 | *A. terreus* | *A. terreus* | NA | 1 | M | General (surgical) | 2023 | Probable |
| TMML 4104 | *A. fumigatus* | *A. fumigatiaffinis* | NA | 1 | F | General | 2023 | Possible |
| TMML 4105 | *A. flavus* | *A. flavus* | NA | 2 | M | ICU | 2023 | Probable |
| TMML 4106 | *A. flavus* | *A. flavus* | NA | 2 | M | ICU | 2023 | Probable |
| TMML 4111 | *A. flavus* | *A. flavus* | NA | 1 | M | General | 2023 | Colonization |
| TMML 4113 | *A. flavus* | *A. flavus* | NA | 1 | F | General | 2023 | Probable |
| TMML 4129 | *A. flavus* | *A. flavus* | NA | 2 | M | ENT | 2023 | Proven |
| TMML 4130 | *A. flavus* | *A. flavus* | NA | 2 | F | ENT | 2023 | Proven |
| TMML 4131 | *A. flavus* | *A. flavus* | NA | 2 | F | ENT | 2023 | Proven |
| TMML 4148 | *A. flavus* | *A. flavus* | NA | 2 | F | ENT | 2023 | Proven |
| TMML 49 | *A. flavus* | *A. flavus* | BAL | 1 | F | Hematology | 2023 | Proven |
| TMML 563 | *A. fumigatus* | *A. fumigatus* | Psudomycetoma, Foot | 2 | F | General | 2023 | Probable |
| TMML 59 | *A. flavus* | *A. flavus* | BAL | 1 | M | ICU | 2023 | Proven |
| TMML 630 | *A. flavus* | *A. flavus* | Nose contents | 3 | F | General (surgical) | 2021 | Possible |
| TMML 79 | *A. flavus* | *A. flavus* | Sinus tissue | 5 | M | ENT | 2023 | Colonization |
| TMML 87 | *A. flavus* | *A. flavus* | BAL | 1 | F | ICU general | 2023 | Probable |
| TMML 88 | *A. flavus* | *A. flavus* | Sinus | 1 | M | General | 2022 | Proven |
| TMML 89 | *A. flavus* | *A. flavus* | Nasal contents | 1 | M | General | 2020 | Possible |

BAL, bronchoalvealar lavage; ID, infectious; M, male; F, female; ICU, intensive care unit; NA, not available; ENT, ear nose and throat.

**Supplementary table 3: *In vitro* antifungal minimum inhibitory concentrations (MICs) and minimum effective concentrations (MECs) according to CLSI M38 guidelines of 127 *Aspergillus* isolates and corresponding calmodulin (*CaM*) accession numbers in GenBank.** MICs and MECs in µg/mL.

| **ID** | **Species** | **AMB** | **VOR** | **ITC** | **POS** | **ISA** | **AFG** | **MFG** | ***CaM*** |  |
| --- | --- | --- | --- | --- | --- | --- | --- | --- | --- | --- |
| TMML 049 | *A. flavus* | 0.5 | 0.5 | 0.25 | 0.063 | 0.5 | ≤0.008 | ≤0.008 | PP350408 |  |
| TMML 059 | *A. flavus* | 1 | 0.5 | 0.125 | 0.063 | 0.5 | ≤0.008 | ≤0.008 | PP350409 |  |
| TMML 079 | *A. flavus* | 1 | 1 | 0.25 | 0.063 | 0.5 | ≤0.008 | ≤0.008 | PP350410 |  |
| TMML 087 | *A. flavus* | 1 | 1 | 0.125 | 0.063 | 1 | ≤0.008 | ≤0.008 | PP350317 |  |
| TMML 088 | *A. flavus* | 0.5 | 0.5 | 0.125 | 0.031 | 0.5 | ≤0.008 | ≤0.008 | PP350318 |  |
| TMML 089 | *A. flavus* | 1 | 0.5 | 0.25 | 0.063 | 0.25 | ≤0.008 | ≤0.008 | PP350319 |  |
| TMML 101 | *A. terreus* | 0.5 | 0.25 | 0.125 | 0.031 | 0.25 | ≤0.008 | ≤0.008 | PP350320 |  |
| TMML 102 | *A. flavus* | 0.5 | 1 | 0.25 | 0.125 | 1 | ≤0.008 | ≤0.008 | PP350321 |  |
| TMML 104 | *A. flavus* | 0.5 | 0.5 | 0.125 | 0.063 | 0.5 | ≤0.008 | ≤0.008 | PP350322 |  |
| TMML 105 | *A. flavus* | 2 | 0.5 | 0.25 | 0.063 | 0.5 | ≤0.008 | ≤0.008 | PP350323 |  |
| TMML 106 | *A. fumigatus* | 0.25 | 0.25 | 0.25 | 0.031 | 0.25 | ≤0.008 | ≤0.008 | PP350324 |  |
| TMML 107 | *A. flavus* | 1 | 1 | 0.125 | 0.063 | 0.5 | ≤0.008 | ≤0.008 | PP350325 |  |
| TMML 108 | *A. flavus* | 0.5 | 0.5 | 0.25 | 0.063 | 0.25 | ≤0.008 | ≤0.008 | PP350326 |  |
| TMML 563 | *A. fumigatus* | 0.25 | 0.125 | 0.125 | 0.031 | 0.125 | ≤0.008 | ≤0.008 | PP350407 |  |
| TMML 630 | *A. flavus* | 1 | 0.5 | 0.125 | 0.063 | 0.25 | ≤0.008 | ≤0.008 | PP350327 |  |
| TMML 1609 | *A. flavus* | 0.5 | 1 | 0.25 | 0.063 | 0.5 | ≤0.008 | ≤0.008 | PP350328 |  |
| TMML 2122 | *A. flavus* | 2 | 0.5 | 0.25 | 0.063 | 0.25 | ≤0.008 | ≤0.008 | PP350329 |  |
| TMML 2155 | *A. fumigatus* | 0.25 | 1 | 0.25 | 0.063 | 0.5 | ≤0.008 | ≤0.008 | PP350330 |  |
| TMML 2156 | *A. fumigatus* | 0.125 | 0.5 | 0.25 | 0.031 | 0.25 | ≤0.008 | ≤0.008 | PP350331 |  |
| TMML 2277 | *A. fumigatus* | 0.25 | 0.5 | 0.25 | 0.063 | 0.5 | ≤0.008 | ≤0.008 | PP350332 |  |
| TMML 2278 | *A. flavus* | 1 | 0.5 | 0.25 | 0.063 | 0.5 | ≤0.008 | ≤0.008 | PP350333 |  |
| TMML 2282 | *A. flavus* | 1 | 1 | 0.125 | 0.125 | 0.5 | ≤0.008 | ≤0.008 | PP350334 |  |
| TMML 2293 | *A. flavus* | 1 | 0.5 | 0.125 | 0.063 | 1 | ≤0.008 | ≤0.008 | PP350335 |  |
| TMML 2302 | *A. fumigatus* | 0.25 | 0.25 | 0.25 | 0.031 | 0.25 | 0.016 | ≤0.008 | PP350336 |  |
| TMML 2317 | *A. niger* | 0.125 | 0.25 | 0.25 | 0.125 | 0.5 | ≤0.008 | ≤0.008 | PP350337 |  |
| TMML 2318 | *A. niger* | 0.125 | 0.5 | 0.5 | 0.063 | 0.5 | ≤0.008 | ≤0.008 | PP350338 |  |
| TMML 2329 | *A. fumigatus* | 0.25 | 1 | 0.25 | 0.031 | 1 | ≤0.008 | ≤0.008 | PP350339 |  |
| TMML 2367 | *A. fumigatus* | 0.25 | 0.25 | 0.125 | 0.031 | 0.25 | ≤0.008 | ≤0.008 | PP350340 |  |
| TMML 2375 | *A. fumigatus* | 0.25 | 0.5 | 0.063 | 0.063 | 0.5 | ≤0.008 | ≤0.008 | PP350341 |  |
| TMML 2379 | *A. niger* | 0.125 | 0.25 | 0.125 | 0.031 | 0.25 | ≤0.008 | ≤0.008 | PP350342 |  |
| TMML 2397 | *A. fumigatus* | 0.25 | 0.5 | 0.25 | 0.031 | 0.5 | ≤0.008 | ≤0.008 | PP350343 |  |
| TMML 2399 | *A. flavus* | 1 | 1 | 0.5 | 0.063 | 1 | ≤0.008 | ≤0.008 | PP350344 |  |
| TMML 2403 | *A. flavus* | 1 | 1 | 0.125 | 0.063 | 0.5 | ≤0.008 | ≤0.008 | PP350345 |  |
| TMML 2405 | *A. flavus* | 0.5 | 0.25 | 0.125 | 0.063 | 0.25 | ≤0.008 | ≤0.008 | PP350346 |  |
| TMML 2406 | *A. flavus* | 0.5 | 1 | 0.25 | 0.125 | 0.5 | ≤0.008 | ≤0.008 | PP350347 |  |
| TMML 2410 | *A. flavus* | 1 | 0.5 | 0.125 | 0.063 | 0.25 | ≤0.008 | 0.016 | PP350348 |  |
| TMML 2411 | *A. fumigatus* | 0.25 | 1 | 0.5 | 0.063 | 0.5 | ≤0.008 | ≤0.008 | PP350349 |  |
| TMML 2429 | *A. flavus* | 1 | 0.5 | 0.25 | 0.063 | 0.25 | ≤0.008 | ≤0.008 | PP350350 |  |
| TMML 2432 | *A. flavus* | 1 | 1 | 0.125 | 0.125 | 1 | ≤0.008 | ≤0.008 | PP350351 |  |
| TMML 2456 | *A. flavus* | 1 | 0.25 | 0.125 | 0.063 | 0.5 | ≤0.008 | ≤0.008 | PP350352 |  |
| TMML 2457 | *A. flavus* | 2 | 1 | 0.25 | 0.063 | 1 | ≤0.008 | ≤0.008 | PP350353 |  |
| TMML 2471 | *A. niger* | 0.063 | 0.5 | 0.5 | 0.125 | 0.5 | ≤0.008 | ≤0.008 | PP350354 |  |
| TMML 2472 | *A. flavus* | 0.5 | 0.5 | 0.125 | 0.063 | 0.25 | ≤0.008 | 0.016 | PP350355 |  |
| TMML 2473 | *A. flavus* | 1 | 0.5 | 0.25 | 0.063 | 0.25 | ≤0.008 | ≤0.008 | PP350356 |  |
| TMML 2474 | *A. flavus* | 1 | 0.5 | 0.125 | 0.063 | 0.25 | ≤0.008 | ≤0.008 | PP350357 |  |
| TMML 2479 | *A. flavus* | 0.5 | 0.25 | 0.25 | 0.063 | 0.25 | 0.016 | 0.016 | PP350358 |  |
| TMML 2482 | *A. flavus* | 0.5 | 1 | 0.125 | 0.125 | 1 | ≤0.008 | ≤0.008 | PP350359 |  |
| TMML 2483 | *A. terreus* | 0.25 | 0.25 | 0.125 | 0.031 | 0.25 | 0.016 | ≤0.008 | PP350360 |  |
| TMML 2486 | *A. flavus* | 1 | 0.5 | 0.063 | 0.063 | 0.5 | ≤0.008 | ≤0.008 | PP350361 |  |
| TMML 2487 | *A. flavus* | 4 | 1 | 0.125 | 0.125 | 0.5 | ≤0.008 | ≤0.008 | PP350362 |  |
| TMML 2496 | *A. flavus* | 0.5 | 1 | 0.125 | 0.125 | 1 | ≤0.008 | ≤0.008 | PP350363 |  |
| TMML 2497 | *A. terreus* | 0.25 | 0.25 | 0.031 | 0.031 | 0.25 | ≤0.008 | ≤0.008 | PP350364 |  |
| TMML 2498 | *A. terreus* | 0.25 | 1 | 0.125 | 0.063 | 0.5 | ≤0.008 | ≤0.008 | PP350365 |  |
| TMML 2504 | *A. terreus* | 0.25 | 0.25 | 0.25 | 0.031 | 0.25 | ≤0.008 | ≤0.008 | PP350366 |  |
| TMML 2505 | *A. flavus* | 1 | 1 | 0.125 | 0.125 | 1 | ≤0.008 | ≤0.008 | PP350367 |  |
| TMML 2507 | *A. fumigatus* | 0.5 | 0.25 | 0.125 | 0.063 | 0.25 | ≤0.008 | ≤0.008 | PP350368 |  |
| TMML 2508 | *A. flavus* | 0.5 | 0.25 | 0.063 | 0.063 | 0.5 | ≤0.008 | ≤0.008 | PP350369 |  |
| TMML 2516 | *A. niger* | 0.125 | 0.25 | 0.25 | 0.063 | 0.25 | ≤0.008 | ≤0.008 | PP350370 |  |
| TMML 2522 | *A. flavus* | 0.5 | 1 | 0.25 | 0.063 | 0.5 | ≤0.008 | ≤0.008 | PP350371 |  |
| TMML 2524 | *A. citrinoterreus* | 1 | 0.25 | ≤0.016 | ≤0.016 | 0.125 | ≤0.008 | ≤0.008 | PP350372 |  |
| TMML 2535 | *A. terreus* | 0.5 | 0.5 | 0.25 | 0.063 | 0.25 | ≤0.008 | ≤0.008 | PP350373 |  |
| TMML 2538 | *A. flavus* | 1 | 0.25 | 0.125 | 0.031 | 0.25 | ≤0.008 | ≤0.008 | PP350374 |  |
| TMML 2539 | *A. tubingensis* | 0.125 | 1 | 0.25 | 0.063 | 1 | ≤0.008 | ≤0.008 | PP350375 |  |
| TMML 2540 | *A. flavus* | 1 | 0.5 | 0.5 | 0.063 | 0.25 | 0.016 | ≤0.008 | PP350376 |  |
| TMML 2541 | *A. flavus* | 1 | 1 | 0.125 | 0.063 | 1 | ≤0.008 | ≤0.008 | PP350377 |  |
| TMML 2554 | *A. flavus* | 0.5 | 0.5 | 0.125 | 0.063 | 0.5 | ≤0.008 | ≤0.008 | PP350378 |  |
| TMML 2561 | *A. flavus* | 1 | 0.5 | 0.25 | 0.063 | 0.5 | ≤0.008 | ≤0.008 | PP350379 |  |
| TMML 2565 | *A. fumigatus* | 0.25 | 0.5 | 0.25 | 0.063 | 0.5 | 0.016 | ≤0.008 | PP350380 |  |
| TMML 2566 | *A. flavus* | 1 | 0.5 | 0.5 | 0.063 | 1 | ≤0.008 | ≤0.008 | PP350381 |  |
| TMML 2567 | *A. flavus* | 1 | 1 | 0.063 | 0.063 | 0.5 | ≤0.008 | ≤0.008 | PP350382 |  |
| TMML 2575 | *A. flavus* | 1 | 0.5 | 0.25 | 0.063 | 0.5 | ≤0.008 | ≤0.008 | PP350383 |  |
| TMML 2594 | *A. flavus* | 1 | 0.5 | 0.25 | 0.063 | 0.5 | ≤0.008 | ≤0.008 | PP350384 |  |
| TMML 2636 | *A. flavus* | 0.5 | 1 | 0.25 | 0.125 | 1 | ≤0.008 | ≤0.008 | PP350385 |  |
| TMML 2640 | *A. flavus* | 0.5 | 0.5 | 0.125 | 0.063 | 0.5 | ≤0.008 | ≤0.008 | PP350386 |  |
| TMML 3613 | *A. niger* | 0.125 | 0.25 | 0.125 | 0.125 | 0.25 | ≤0.008 | ≤0.008 | PP350387 |  |
| TMML 3640 | *A. flavus* | 1 | 1 | 0.25 | 0.063 | 0.5 | ≤0.008 | ≤0.008 | PP350388 |  |
| TMML 3641 | *A. flavus* | 0.5 | 0.25 | 0.125 | 0.031 | 0.25 | ≤0.008 | ≤0.008 | PP350389 |  |
| TMML 3644 | *A. flavus* | 1 | 0.5 | 0.25 | 0.063 | 0.5 | ≤0.008 | 0.016 | PP350390 |  |
| TMML 3645 | *A. flavus* | 1 | 1 | 0.25 | 0.125 | 1 | ≤0.008 | ≤0.008 | PP350391 |  |
| TMML 3663 | *A. flavus* | 1 | 1 | 0.063 | 0.063 | 1 | ≤0.008 | ≤0.008 | PP350392 |  |
| TMML 3665 | *A. flavus* | 0.5 | 0.5 | 0.125 | 0.063 | 0.5 | ≤0.008 | ≤0.008 | PP350393 |  |
| TMML 3666 | *A. flavus* | 1 | 0.5 | 0.25 | 0.063 | 0.25 | ≤0.008 | ≤0.008 | PP350394 |  |
| TMML 3669 | *A. flavus* | 1 | 0.5 | 0.125 | 0.063 | 0.5 | ≤0.008 | ≤0.008 | PP350395 |  |
| TMML 3680 | *A. flavus* | 1 | 1 | 0.125 | 0.063 | 1 | ≤0.008 | ≤0.008 | PP350396 |  |
| TMML 3701 | *A. flavus* | 0.5 | 0.5 | 0.125 | 0.031 | 0.5 | ≤0.008 | ≤0.008 | PP350397 |  |
| TMML 3706 | *A. flavus* | 0.5 | 1 | 0.125 | 0.063 | 1 | ≤0.008 | ≤0.008 | PP350398 |  |
| TMML 3768 | *A. flavus* | 0.5 | 1 | 0.25 | 0.063 | 1 | ≤0.008 | ≤0.008 | PP350399 |  |
| TMML 3781 | *A. flavus* | 1 | 0.5 | 0.125 | 0.125 | 0.5 | ≤0.008 | ≤0.008 | PP350400 |  |
| TMML 3785 | *A. candidus* | 0.25 | 0.25 | 0.25 | 0.031 | 0.25 | ≤0.008 | ≤0.008 | PP350401 |  |
| TMML 3787 | *A. flavus* | 0.5 | 1 | 0.125 | 0.063 | 0.5 | ≤0.008 | ≤0.008 | PP350402 |  |
| TMML 3805 | *A. flavus* | 0.5 | 0.5 | 0.125 | 0.063 | 0.5 | ≤0.008 | ≤0.008 | PP350403 |  |
| TMML 3809 | *A. flavus* | 0.5 | 1 | 0.125 | 0.125 | 1 | ≤0.008 | ≤0.008 | PP350404 |  |
| TMML 3822 | *A. flavus* | 0.5 | 1 | 0.25 | 0.125 | 1 | ≤0.008 | ≤0.008 | PP350405 |  |
| TMML 3846 | *A. fumigatus* | 0.25 | 1 | 0.25 | 0.125 | 1 | ≤0.008 | ≤0.008 | PP795459 |  |
| TMML 3857 | *A. flavus* | 2 | 0.5 | 0.125 | 0.063 | 0.5 | ≤0.008 | ≤0.008 | PP795460 |  |
| TMML 3868 | *A. flavus* | 1 | 1 | 0.125 | 0.063 | 1 | ≤0.008 | ≤0.008 | PP795461 |  |
| TMML 3869 | *A. flavus* | 0.5 | 1 | 0.25 | 0.125 | 0.5 | ≤0.008 | ≤0.008 | PP795462 |  |
| TMML 3880 | *A. fumigatus* | 0.125 | 0.5 | 0.25 | 0.063 | 0.5 | ≤0.008 | ≤0.008 | PP350406 |  |
| TMML 3889 | *A. flavus* | 0.5 | 1 | 0.125 | 0.063 | 1 | 0.016 | ≤0.008 | PP795463 |  |
| TMML 3891 | *A. flavus* | 1 | 0.5 | 0.25 | 0.063 | 1 | ≤0.008 | ≤0.008 | PP795464 |  |
| TMML 3894 | *A. flavus* | 1 | 1 | 0.125 | 0.125 | 0.5 | ≤0.008 | ≤0.008 | PP795465 |  |
| TMML 3898 | *A. flavus* | 0.5 | 0.5 | 0.125 | 0.063 | 0.5 | ≤0.008 | ≤0.008 | PP795466 |  |
| TMML 3901 | *A. flavus* | 2 | 0.25 | 0.063 | 0.031 | 1 | ≤0.008 | 0.016 | PP795467 |  |
| TMML 3903 | *A. flavus* | 0.5 | 0.5 | 0.125 | 0.031 | 0.25 | ≤0.008 | ≤0.008 | PP795468 |  |
| TMML 3940 | *A. flavus* | 0.5 | 1 | 0.063 | 0.125 | 1 | ≤0.008 | ≤0.008 | PP795469 |  |
| TMML 3969 | *A. flavus* | 1 | 1 | 0.125 | 0.063 | 0.5 | ≤0.008 | 0.016 | PP795470 |  |
| TMML 3980 | *A. fumigatus* | 0.25 | 1 | 0.125 | 0.063 | 0.5 | ≤0.008 | ≤0.008 | PP795471 |  |
| TMML 3983 | *A. flavus* | 2 | 0.25 | 0.25 | 0.125 | 0.25 | ≤0.008 | ≤0.008 | PP795472 |  |
| TMML 3994 | *A. flavus* | 1 | 0.5 | 0.063 | 0.031 | 1 | ≤0.008 | ≤0.008 | PP795473 |  |
| TMML 3998 | *A. flavus* | 1 | 1 | 0.125 | 0.063 | 1 | ≤0.008 | 0.016 | PP795474 |  |
| TMML 4003 | *A. flavus* | 1 | 0.25 | 0.125 | 0.063 | 0.25 | ≤0.008 | ≤0.008 | PP795475 |  |
| TMML 4008 | *A. flavus* | 0.5 | 1 | 0.25 | 0.125 | 0.25 | 0.016 | ≤0.008 | PP795476 |  |
| TMML 4042 | *A. flavus* | 0.5 | 1 | 0.125 | 0.031 | 0.5 | ≤0.008 | ≤0.008 | PP795478 |  |
| TMML 4066 | *A. flavus* | 1 | 0.25 | 0.25 | 0.063 | 0.5 | ≤0.008 | ≤0.008 | PP795479 |  |
| TMML 4068 | *A. fumigatus* | 0.125 | 0.5 | 0.125 | 0.125 | 0.25 | 0.016 | ≤0.008 | PP795480 |  |
| TMML 4081 | *A. flavus* | 2 | 1 | 0.125 | 0.063 | 1 | ≤0.008 | ≤0.008 | PP795481 |  |
| TMML 4102 | *A. flavus* | 1 | 1 | 0.063 | 0.063 | 0.5 | ≤0.008 | 0.016 | PP795482 |  |
| TMML 4103 | *A. terreus* | 1 | 1 | 0.25 | 0.031 | 0.25 | ≤0.008 | ≤0.008 | PP795483 |  |
| TMML 4104 | *A. fumigatiaffinis* | 1 | 2 | 1 | 0.125 | 1 | ≤0.008 | ≤0.008 | PP795484 |  |
| TMML 4105 | *A. flavus* | 2 | 0.5 | 0.25 | 0.125 | 1 | ≤0.008 | ≤0.008 | PP795485 |  |
| TMML 4106 | *A. flavus* | 0.5 | 1 | 0.125 | 0.063 | 0.5 | ≤0.008 | ≤0.008 | PP795486 |  |
| TMML 4111 | *A. flavus* | 0.5 | 0.5 | 0.25 | 0.031 | 0.5 | ≤0.008 | ≤0.008 | PP795487 |  |
| TMML 4113 | *A. flavus* | 0.5 | 0.25 | 0.125 | 0.063 | 2 | 0.016 | ≤0.008 | PP795488 |  |
| TMML 4129 | *A. flavus* | 1 | 1 | 0.063 | 0.125 | 1 | ≤0.008 | ≤0.008 | PP795489 |  |
| TMML 4130 | *A. flavus* | 1 | 0.5 | 0.125 | 0.063 | 0.5 | 0.016 | ≤0.008 | PP795490 |  |
| TMML 4131 | *A. flavus* | 0.5 | 1 | 0.063 | 0.031 | 1 | ≤0.008 | ≤0.008 | PP795491 |  |
| TMML 4148 | *A. flavus* | 2 | 1 | 0.125 | 0.125 | 1 | ≤0.008 | ≤0.008 | PP795492 |  |

AMB, amphotericin B; VOR, voriconazole; ITC, itraconazole; POS, posaconazole; ISA, isavuconazole; AFG, anidulafungin; MFG, micafungin.
